# Selenium and Skeletal Muscle Health in Sports Nutrition

**DOI:** 10.3390/nu17111902

**Published:** 2025-05-31

**Authors:** Qi Wang, Jiaqiang Huang, Kongdi Zhu, Wei Zuo

**Affiliations:** 1 Institute of Artificial Intelligence in Sports, Capital University of Physical Education and Sports, Beijing 100083, China; 00020240005@cupes.edu.cn; 2 Beijing Advanced Innovation Center for Food Nutrition and Human Health, Department of Nutrition and Health, China Agricultural University, Beijing 100083, China; jqhuang@cau.edu.cn; 3 Key Laboratory of Precision Nutrition and Food Quality, Ministry of Education, Department of Nutrition and Health, China Agricultural University, Beijing 100083, China

**Keywords:** selenium, selenoprotein, skeletal muscle health

## Abstract

Selenium is a trace element of fundamental importance to human health. In recent years, an increasing number of studies have been carried out in the field of skeletal muscle health and sports nutrition. Selenium functions in the human body through selenoproteins. Selenoproteins play an important role in maintaining skeletal muscle function by delaying exercise fatigue and muscle aging. They mainly regulate skeletal muscle by anti-oxidation, regulating signal pathways, and affecting protein metabolism. In this paper, we summarize the latest advancements in research regarding selenium and its impact on skeletal muscle health, along with its applications in sports nutrition.

## 1. Introduction

Selenium functions in the body mainly through selenoproteins. Selenoproteins are a special protein family containing the 21st amino acid (selenocysteine, Sec) [1,2]. Sec is translated by the UGA stop codon and inserted into the protein. In eukaryotes, the SEC-insertion sequence (SECIS) element located in the 3′-untranslated region (3′UTR) of selenoprotein mRNA distinguishes between the UGA codon used to translate Sec and the UGA stop codon and recognizes the binding of SECIS binding protein (SBP2) to it [3,4]. Thus, selenoprotein synthesis can be completed. So far, 25 selenium proteins have been found in humans [5]. These selenoproteins have a variety of biological functions—including regulation of thyroid hormone metabolism, intracellular and extracellular antioxidation, reverse transport of proteins from endoplasmic reticulum to cytoplasm, etc.—which play an important role in maintaining the homeostasis of cells and tissues and the health of the body [6]. Se deficiency in individuals may cause a variety of diseases, such as tissue oxidative stress, white muscle disease, and even cancer.

Skeletal muscle is a main target organ for tissue damage caused by Se deficiency. The skeletal muscle tissue will be dysfunctional when organisms are deficient in selenium. Se deficiency may eventually lead to impaired muscle contraction, muscle necrosis, and atrophy, due to vascular injury, degeneration, and extensive calcification [7,8]. Selenium deficiency affects the recovery of muscle atrophy and directly causes metabolic disorder in skeletal muscle, which, in turn, affects the normal physiological function of skeletal muscle [9]. Skeletal muscle represents the most massive tissue in the body in adult mammals [10]. Following injury, skeletal muscle displays a powerful regenerative capacity that is attributed to the ordered regulation of a cascade of events initiated by satellite cells (SCs). Skeletal muscle is indispensable for critical physiological functions, including locomotion, postural stabilization, respiratory mechanics, and thermoregulation. This tissue predominantly consists of multinucleated myofibers formed through mitotic fusion during development. The homeostatic maintenance and repair of muscle tissue are facilitated by resident SCs, which reside in a quiescent state between the basal lamina and sarcolemma of myofibers. Under physiological conditions, SCs remain primed for activation in response to mechanical injury or exogenous growth signals, initiating myogenic differentiation and regeneration processes to preserve tissue integrity [11]. Emerging evidence highlights the critical importance of selenium and selenoproteins in skeletal muscle biology, particularly their roles in reducing exercise-induced fatigue, improving post-exercise recovery, and combating age-related muscle decline. However, the comprehensive mechanisms through which Se and selenoproteins maintain skeletal muscle health remain to be fully elucidated. This review systematically consolidates current knowledge regarding selenium’s biological mechanisms, focusing on three key aspects, i.e., (1) antioxidant regulation, (2) mitochondrial function modulation, and (3) protein metabolism regulation, while discussing their practical applications for enhancing athletic performance and maintaining muscle health.

## 2. Selenoproteins in Skeletal Muscle Biology

**Figure 1:**
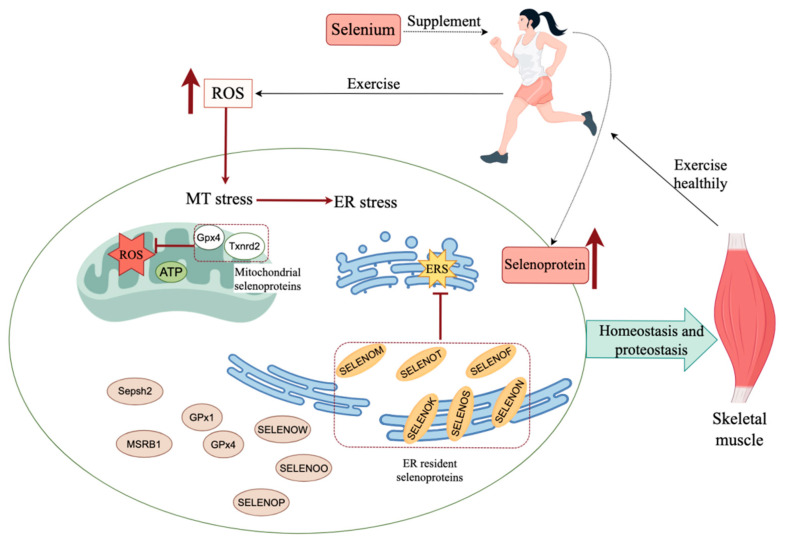
Synergistic effects of selenoprotein networks in skeletal muscle. Several key selenoproteins collaborate to eliminate excess ROS, alleviate mitochondrial and ER stress, and restore cellular homeostasis disrupted by oxidative stress induced by excessive exercise. Moderate selenium supplementation helps maintain skeletal muscle homeostasis and protease balance, thereby enhancing exercise performance and fitness. ER: endoplasmic reticulum; ERS: endoplasmic reticulum stress; ROS: reactive oxygen species; MT:mitochondria.The red up thick arrow in the figure indicates the increase in number. The figure was created with https://www.figdraw.com/ (accessed on 10 May 2025).

Se mediates its physiological roles in mammals predominantly through selenoproteins. Of the 25 selenoproteins identified thus far in mammals, two (GPX4 and TXNRD2) are classified as mitochondrial selenoproteins, while seven others (DIO2, SELENOF, SELENOK, SELENOS, SELENOM, SELENON, and SELENOT) are localized in the endoplasmic reticulum [5]. These selenoproteins have the ability to regulate mitochondrial and endoplasmic reticulum (ER) homeostasis. Previous studies have focused on the function of individual selenium proteins. Jing et al. showed that the biological function of selenoproteins is not a single one, but a synergistic function of multiple selenoproteins [12]. We describe the mechanisms by which selenium supplementation exerts its effects in muscle cells in the form of selenoproteins to promote exercise fitness in Figure 1.

### 2.1. SELENOW

Selenoprotein W (SELENOW) is a skeletal muscle-enriched selenoprotein with a thioredoxin-like fold; it belongs to the Rdx family. It was the first selenoprotein described to be linked to white muscle disease in lambs [13]. Numerous studies have demonstrated that SELENOW can function in redox regulation [14,15], cell cycle progression [16], and myogenic differentiation [17,18], which are related to muscle growth and development. It also plays a pivotal role in calcium homeostasis and proteostasis. During selenium deficiency, downregulation of SELENOW disrupts the STIM1/TRPC1-mediated calcium influx, leading to impaired myoblast fusion and muscle regeneration. In dexamethasone-induced atrophy models, SELENOW knockout mice exhibited exacerbated muscle loss via suppression of RAC1-mTOR signaling and activation of ubiquitin ligases (MuRF1/Atrogin-1) [19]. Clinically, SELENOW expression is positively correlated with grip strength in elderly populations, suggesting its potential as a biomarker for sarcopenia [20].

SELENOW also plays a pivotal role in redox regulation through its interaction with GSH [19]. Mechanistically, under oxidative stress, glutathione S-transferase Pi (GSTP1) catalyzes the S-glutathionylation of murine SELENOW at cysteine residue 33 (Cys33). This post-translational modification prevents the formation of a second disulfide bond within SELENOW, thereby enhancing its capacity to mitigate oxidative damage and maintain cellular viability [15].

### 2.2. SELENON

Selenoprotein N (SELENON) is an ER protein whose loss of function leads to human SELENON-related myopathies [21]. It has a thioredoxin reductase-like domain on the ER side of its sequence and regulates ER calcium levels by means of the redox regulating SERCA2 pump [22]. SELENON directly interacts with ryanodine receptors (RyRs), constituting critical components of calcium release channels which serve as molecular regulators of endoplasmic reticulum calcium homeostasis and redox equilibrium [23,24,25]. SELENON is a selenoprotein that is widely recognized to play an important role in skeletal muscle diseases. In the presence of selenium deficiency, decreased SELENON content leads to decreased calcium release from the sarcoplasmic reticulum and increased production of tonic contraction-associated ROS, directly leading to skeletal muscle pain, fatigue, and proximal weakness that contribute to polyaxial disease, congenital muscular dystrophy, and sarcopenia during aging, changes that can be alleviated by selenium, and sarcopenia during aging, changes that can be alleviated by selenium supplementation [26,27].

### 2.3. SELENOK

Selenoprotein K (SELENOK) is also an ER protein. It is abundantly expressed in muscle tissue and has functions such as the degradation of ER-related proteins, anti-oxidation, regulation of Ca^2+^ flux in the ER, and participation in immune response. Previous studies have shown that SELENOK knockdown can cause an imbalance in calcium homeostasis and glucose metabolism in chick embryo myoblasts, as well as impairing muscle development. Studies have shown that SELENOK silencing impairs skeletal muscle repair and inhibits myogenic differentiation of satellite cells. SELENOK plays a key role in regulating the homeostasis of anti-oxidation and ER stress in myoblasts, as well as the level of apoptosis and autophagy. Therefore, SELENOK is involved in the mechanism of satellite cell-led skeletal muscle regeneration. SELENOK binds to a variety of chaperones (p97, SELENOS, Derlin1) on the ER and regulates the intensity of UPR response by promoting ERAD to alleviate ER stress. SELENOK can regulate ER stress by promoting ERAD during skeletal muscle regeneration and regulate antioxidant signaling in an ER stress-dependent manner during myoblast differentiation. Selenoprotein K pro-tects skeletal muscle from damage and is required for SC-mediated myogenic differen-tiation [28,29].

### 2.4. SELENOS

Selenoprotein S (SELENOS), highly expressed in skeletal muscle, is a transmembrane selenoprotein that resides in the ER and has a C-terminal selenocysteine residue with antioxidant properties and the ability to confer protection against ER stress [30]. In vitro studies have shown that decreased expression of SELENOS (SELENOS) increases oxidative stress and ER stress in various mammalian cell lines [31,32,33]. Studies have found that SEPS1 has a protective and anti-inflammatory effect in vivo in a lipopolysaccharide (LPS)-induced sepsis mouse model [34]. Genetic polymorphisms of SELENOS are associated with elevated proinflammatory cytokines, and SELENOS has a protective effect against inflammatory stress in vitro. SELENOS polymorphisms can cause congenital muscular dystrophy [35]. Recent studies have identified SELENOS as a novel disease-modifying gene in muscular dystrophy and myopathy by investigating whether genetic depletion of SELENOS in mdx-dystrophic mice exacerbates skeletal muscle inflammation and impairs the structure and function of dystrophic hind limb muscles [36].

### 2.5. GPx

One of the primary antioxidant systems in cells is catalyzed by the selenoenzyme Glutathione peroxidase (GPx) [37]. The main function of the GPx family is to scavenge ROS and protect cell membrane integrity. GPx1 plays a role in anti-oxidation in the cytoplasm and reducing lipid peroxidation after exercise. GPx4, localized in mitochondria, uniquely prevents ferroptosis by reducing lipid hydroperoxides [19]. In endurance athletes, the activity of muscle GPx increases by 65% post-training, protecting mitochondrial membranes from oxidative rupture [38,39]. Conversely, GPx4 inhibition triggers iron-dependent lipid peroxidation, causing necrotic myofiber death in selenium-deficient models [40].

### 2.6. Txnrd

Thioredoxin reductase (Txnrd) is an oxidoreductase that catalyzes the reduction of oxidized Thioredoxin (Trx) using NADPH [5]. Trx is, in turn, used by some cellular enzymes as a cofactor in dithio-disulfide bond exchange reactions, which are the main intracellular mechanism for maintaining a reducing environment, especially for the maintenance of reduced cysteine groups. Mammals express three Txnrd isoforms: Txnrd1 (cytoplasmic/nuclear; TR1/TrxR1), responsible for reducing cytosolic thioredoxin (Trx1); Txnrd2 (mitochondrial; TR3/TrxR2), which reduces mitochondrial thioredoxin (Trx2); and Txnrd3 (testis-specific; not discussed here). Notably, cardiomyocyte-specific deletion studies revealed that Txnrd2 is indispensable for viability, underscoring its non-redundant role in maintaining mitochondrial redox homeostasis during cardiac development. This tissue-specific essentiality likely stems from the high energetic demands and ROS exposure inherent to myocardial cells, necessitating robust Txnrd2-mediated antioxidant protection within mitochondria. Based on existing studies, research on the mechanism of TXNRD1 function in sport related fields mainly lies in animal and in vitro verification [41,42,43,44,45].

### 2.7. MsrB1

Methionine r-sulfoxide reductase (MsrB1) is the main mammalian MsrB located in the cytosol and nucleus. MSRB1 was found to control mammalian actin assembly and breakdown. Micals have been the only known partners of MsrB1, and actin is the only target. This suggests that MSRB1 may be involved in skeletal muscle growth regulation [46]. In addition, MSRB1 is involved in the regulation of redox homeostasis and protects proteins from oxidative damage [47].

### 2.8. Other Selenoproteins

In addition to the selenoproteins described above, there are a number of selenoproteins that may play a role in maintaining muscle health status. Selenoprotein P (SELENOP) is mainly responsible for the transport of Se from plasma to various target organs, thus controlling the expression of all selenoproteins and SEPHS2 and providing systemic antioxidant defense [12]. Selenoprotein O (SELENOO) is the largest mammalian selenoprotein. SELENOO has been shown to engage in redox interactions with unknown proteins via its CXXU motif. Redox regulation of protein function in mitochondria may involve kinase function [1]. As an ER selenoprotein, Selenoprotein F (SELENOF) can be regulated by both ER stress and selenium status. It may have a functional link with endoplasmic reticulum protein folding and secretion processes. Selenoprotein F (SELENOF) interacts with UDP-glucose:glycoprotein glucosyltransferase (UGGT) to participate in ER quality control mechanisms, ensuring proper folding of nascent glycoproteins. Following successful folding, these proteins are packaged into COPII-coated transport vesicles for anterograde trafficking to the Golgi apparatus, where they undergo post-translational modifications prior to secretion via exocytic pathways [48]. SELENOS is a core component of the reverse translocation channel in ER-related protein degradation, which removes misfolded peptide chains and maintains ER homeostasis. Selenoprotein T (SELENOT) is a member of the thioredoxin-like superfamily containing the CXXU redox-active motif. It mainly has the function of maintaining ER redox balance and regulating autophagy and apoptosis. SELENOT can alleviate ER stress and protect myoblast survival by inhibiting the CHOP/ATF4 pathway. SELENOT knockout exacerbates skeletal muscle aplasia [8]. SELENOK, SELENOT, and SELENOM control ER homeostasis by scavenging excess ROS and inhibiting apoptosis [49,50,51,52]. Taking the above analyses together, a summary of the functions of skeletal muscle-associated seloproteins is presented in Table 1. The occurrence of muscle-related diseases is not only related to a single protein but may be caused by multiple proteins together. However, current research is limited, and more in-depth molecular mechanism studies are needed to explore the connection between selenoproteins and muscle-related diseases.

## 3. Mechanisms of Selenium in Muscle Health

Mammalian skeletal muscle accounts for 30–40% of the total body weight and has important physiological functions, including internal organ protection, protein storage, body movement, and thermogenesis [10,73,74]. The physiological hypertrophy of skeletal muscle is characterized by the coordinated accumulation of contractile proteins and limited lipid deposition [75]. Post-exercise oxidative stress (OS) disrupts this process in mammals, resulting in impaired muscle growth through dysregulation of the proteostatic and lipostatic pathways. Mechanistically, OS triggers mitochondrial dysfunction, leading to excessive ROS generation that overwhelms antioxidant defenses. This redox imbalance promotes oxidative modifications of cellular components which concomitantly suppress anabolic signaling while activating catabolic pathways, ultimately driving muscle mass loss [76].

### 3.1. Antioxidant Defense

Exercise-induced oxidative stress is the result of an increase in free radicals produced by mitochondrial respiration that exceeds antioxidant capacity. Selenium is well-regarded for its role in enhancing muscle function, particularly due to its antioxidant properties. Se supplementation may have significant potential in reducing exercise-induced oxidative stress [77]. The GPx family of selenoproteins serves as a pivotal regulator of ROS-mediated oxidative stress, particularly under conditions of heightened metabolic demand or pathological states such as infection or tissue injury. Through enzymatic reduction of hydroperoxides, GPx isoforms maintain redox homeostasis in skeletal muscle, with their activity being strictly dependent on Se incorporation into the catalytic selenocysteine residue. Notably, mitochondrial GPx4 exhibits compartment-specific functionality by preventing lipid peroxidation within inner mitochondrial membranes, thereby preserving electron transport chain integrity. Collectively, Se-dependent antioxidant systems and mitochondrial bioenergetics constitute critical determinants of skeletal muscle redox balance and metabolic plasticity during stress adaptation [57].

### 3.2. Mitochondrial Function

Skeletal muscle is the largest energy-consuming tissue in the body, and mitochondria are the main site of aerobic respiration in cells [78], which, besides being the structure of energy production in cells, play an important role in the differentiation, growth, and development and function of various tissues in the body [79,80] Selenium mainly affects the capacity and function of mitochondria, which, in turn, affects muscle health. Dietary selenium supplementation enhances mitochondrial content in skeletal muscle, as evidenced by elevated mitochondrial volume, density, and respiratory complex activity in both in vivo and in vitro models. SELENOH, SELENON, SELENOW, SELENOO, and DIOs show different effects on mitochondrial and/or skeletal muscle function. SELENOH enhances mitochondrial biogenesis, whereas SELENON and SELENOW appear to affect muscle calcium homeostasis and thus mitochondrial function. In addition, the residence of SELENOO within mitochondria contributes to the redox function of Se. Deiodinase regulates thyroid hormone activation, which, in turn, affects muscle cell regeneration, metabolism, and ROS production [57].

There is also research that shows that the depletion of SELENOT leads to the accumulation of mitochondrial superoxide and downregulation of mitochondrial dynamics gene expression, which, in turn, induces disruption of mitochondrial potential and blocks the oxidative phosphorylation process. Mitochondrial ROS overproduction leads to rate limitation of ATP production, accompanied by cell cycle arrest, slow cell proliferation, and increased myocyte apoptosis. The elimination of mitochondrial ROS has been shown to effectively alleviate the above adverse effects and significantly restore the proliferative potential of myoblasts. Therefore, SELENOT acts as a guardian of cellular homeostasis, resisting mitochondrial oxidative stress, protecting ATP production, promoting myoblast proliferation, and inhibiting apoptosis. Despite this, the exact relationship between dietary Se and skeletal muscle mitochondria is currently unknown [8].

### 3.3. Protein Metabolic Balance

Selenium deficiency disrupts protein turnover by suppressing mTORC1 signaling and activating the ubiquitin-proteasome pathway in skeletal muscle [81]. However, the specific selenoproteins regulating skeletal muscle protein homeostasis remain unclear. The SELENOW-RAC1-mTOR cascade has been proposed as a mechanism coordinating protein synthesis and degradation. In SELENOW-knockout models, reduced EIF4G protein levels and impaired translational activity have been observed. The ubiquitin-proteasome system represents the predominant proteolytic pathway in skeletal muscle [82], driven by the transcription factor FOXO, which upregulates atrophy-associated ubiquitin ligases (Atrogin-1 and MuRF-1) [83]. mTORC2 is essential for AKT-FOXO signaling, while SELENOW modulates both protein synthesis and degradation via the SELENOW-RAC1-mTOR axis, underscoring its critical role in proteostasis [19].

### 3.4. Calcium Homeostasis and Muscle Contraction

Ca^2+^ is a widespread intracellular signal that can participate in the regulation of a variety of cellular physiological processes. Many effectors downstream of the Ca^2+^ signaling pathway are essential for muscle development, including CAM-dependent kinases and phosphatases, mitogen-activated protein kinases (MAPKs), Ca^2+^ -sensitive transcription factors, and nuclear factor of activated T cells (NFATc) [84]. The role of CAM-dependent phosphatases in mouse myogenesis begins with their function in early skeletal muscle cell differentiation by regulating the expression of transcription factors MEF2, MyoD, and myogenin [85]. In addition to the classic CaM dependent way, other signal cascade may help Ca^2+^ dynamics drive the transduction of skeletal muscle development. Signaling through several elements of the MAPK pathway regulates different steps of myogenesis [86,87]. MAPKs and Ca^2+^ signaling in spinal cord [88] can coordinate the regulation of skeletal muscle development [89].

Ca^2+^ signaling is involved in skeletal muscle development and is directly related to the Ca^2+^ + kinetic patterns of developing muscle cell stored Ca^2+^ access (SOCE), prepared by sensors of internal Ca^2+^ stores, STIM1, SOCE channels Orai1, and TRPC channels. STIM1 expression regulates development and peaks in developing mouse muscle [90]. Mice lacking STIM1 die of perinatal skeletal myopathy [91], indicating that STIM1-dependent Ca^2+^ signaling is required for myogenesis. At initiation, TRPC1 expression is also developmentally regulated to differentiate and is required for myoblast migration and fusion into myotubes. SELENOT is involved in the regulation of intracellular calcium concentration in chick embryo myoblasts and significantly affects calcium homeostasis in chick embryo myoblasts. A decrease in SELENOT expression was found to decrease the expression of STIM1 and TRPC1—which are beneficial factors related to muscle development—in calcium channels. Ca^2+^ is an important part of the signal that promotes muscle formation, balance, and regeneration. In particular, changes in Ca^2+^ may directly contribute to muscle satellite cell proliferation or differentiation [9].

## 4. Selenium Deficiency and Muscle Pathologies

Selenium deficiency impairs maximal contractile strength and isokinetic torque in skeletal muscle, resulting in postural instability and motor dysfunction secondary to myopathic pathologies. This deficiency is further characterized by reduced serum creatine kinase and lactate dehydrogenase activity, correlating with clinical manifestations of skeletal muscle pain and fatigue [92]. Previous studies have reported that Se deficiency is the cause of different forms of cardiac and skeletal muscle disease in humans, termed dystrophic muscular dystrophy [93]. These myopathies are characterized by alterations in cardiac or skeletal muscle fibers, resulting in impaired contraction, muscle atrophy, and varying degrees of limb or trunk stiffness. Keshan disease, a selenium-deficient cardiomyopathy, is characterized by myocardial necrosis, inflammatory infiltration, and calcification. This pathology arises from the synergistic effects of dietary selenium insufficiency and coxsackievirus B3 infection. Selenium deficiency impairs selenoenzyme activity (e.g., GPx), disrupting cardiomyocyte redox homeostasis and potentiating viral DNA oxidative damage. The subsequent accumulation of viral genomic mutations enhances virulence, exacerbating myocardial injury [27].

Sarcopenia is closely related to age-related oxidative stress and the imbalance of protein metabolism. Selenium can delay the progression of muscle atrophy by protecting the function of motor neurons and reducing the degeneration of neuromuscular junction. Clinical studies have shown that people with lower plasma selenium levels are more likely to have decreased hip strength and grip strength [20].

## 5. Exercise and Skeletal Muscle Adaptation

### 5.1. Exercise-Induced Increased Selenium Consumption

Intense exercise induces muscle damage, manifesting as delayed-onset muscle soreness and impaired force-generating capacity. This impairment arises from multifactorial mechanisms involving structural disruption of contractile proteins (e.g., myofibrillar Z-disc streaming) and dysregulation of calcium-handling pathways (e.g., sarcoplasmic reticulum Ca^2+^-ATPase dysfunction). These pathophysiological alterations are mediated by mechanical stress from high-intensity eccentric contractions coupled with the exercise-induced overproduction of ROS, which collectively exacerbate sarcolemmal and organelle membrane permeability [94]. Vigorous exercise increases the production of free radicals or ROS and nitrogen (RONS), which hinder muscle contractile function and lead to muscle fatigue and decreased performance [95]. To combat muscle damage and fatigue and improve performance, athletes often take antioxidant supplements [96]. As an antioxidant, selenium has a strong scavenging capacity for ROS and may exert beneficial effects on exercise performance and exercise recovery in athletic populations. At present, some studies have applied selenium in related studies of exercise training, as shown in Table 2. We found limited human studies showing reduced exertion-related lipid peroxidation in overweight participants with low Se levels [77], whereas several studies have shown no beneficial effects on endurance performance [97,98,99]. This may be influenced by the form and dose of selenium consumed, as well as the timing of supplementation.

### 5.2. Effect of Selenium Supplementation on Exercise Recovery

Selenium is an essential trace element; deficiencies arising from dietary insufficiency or malabsorption are associated with multiple disorders in humans and livestock, underscoring its critical physiological role. Furthermore, optimal selenium supplementation is hypothesized to confer benefits across diverse aspects of human health. High-intensity exercise elevates ROS generation, correspondingly increasing selenium-dependent antioxidant demands [12]. Dietary antioxidant supplementation represents a therapeutic strategy to mitigate oxidative damage. Se, a core constituent of endogenous antioxidant systems, scavenges excessive ROS and enhances systemic antioxidant capacity through selenoprotein-mediated redox regulation [105,106]. Despite the benefits of selenium supplementation for muscle health, exercise, especially resistance training, remains central to preventing muscle degeneration. High-intensity resistance training combined with selenium supplementation can significantly improve muscle mass and strength, and the effect is better than that of single intervention.

### 5.3. Dosage and Form of Selenium Supplement

The dose and form of selenium supplements are critical for the effects of selenium. The EFSA has set the dietary reference intake (DRI) of selenium for adults at 70 μg/day and the tolerable upper intake level (UL) at 255 μg/day [107]. The DRI selenium for Chinese residents is 60 μg/day, and the UL is also 400 μg, while the RDI/UL is 55/400 μg per day in US [108]. Selenium levels higher than UL levels result in selenopathy. The main clinical manifestations of this condition are gastrointestinal dysfunction, slow reaction, stiffness of limbs and hair loss. In severe cases, it may cause respiratory failure and death [109]. Therefore, further studies are needed to determine the optimal daily selenium intake for the exercising population and to carry out personalized tailored regimens. Se is supplemented by exogenous intake in humans. It comes from a variety of dietary items including meat, seafood, cereals, dairy products, fruits, vegetables, and some specific Se supplements. Se occurs in many forms in nature. Organic Se (such as selenomethionine and Se yeast) and nano-selenium (SeNPs) have better bioavailability than inorganic Se. Thus, the first two forms of Se should be preferred for the selection of selenium nutritional supplements. In a study of exercising women [110], the consumption of selenium-enriched eggs increased the body’s antioxidant capacity, prevented exercise-induced cellular oxidative damage, and delayed the onset of degenerative diseases. Some animal studies have also found that selenium supplementation is beneficial to the improvement of exercise capacity, as mainly reflected in the improvement of antioxidant capacity. The increase in free radical production and lactate levels induced by acute swimming exercise in rats may be offset by sodium selenite supplementation [111,112]. Se supplementation alleviates liver injury induced by exercise fatigue in rats. Se supplementation can also ameliorate oxidative, energetic, metabolic, and endocrine imbalances via modulation of selenoproteins in rat skeletal muscle [113]. Selenium-rich soybean peptides (SePPs) exhibit higher antioxidant and anti-inflammatory activities in vivo than Selenoprotein, SeMet, sodium selenite (Na_2_SeO_3_), or PPs [114]. (PhSe) 2 was shown to protect against acute physical exercise-induced oxidative damage in mice [115]. SeNPs have also been demonstrated to have tissue antioxidant properties in donkey and grey rabbit, thereby improving exercise fitness [116,117]. However, the optimal timing, dose, and speciation of Se supplementation require further investigation.

## 6. Clinical Implications and Future Directions

### 6.1. Intervention Strategies Targeting Selenoproteins

SELENOP is of significant interest due to its dual role as a biomarker and hepatic-derived transporter to target tissues. SELENOP expression is tightly modulated by dietary selenium availability, inflammatory signals, hypoxic conditions, and pharmacological agents. However, comprehensive pharmacological screening to identify regulatory compounds remains unexplored [118]. Current therapeutic strategies for muscle-related disorders lack targeted delivery systems. Advances in nanotechnology, however, have enabled the development of nanoparticle-based drug carriers (e.g., nanoscale delivery platforms) that demonstrate potential for tissue-specific targeting. Notably, calcium-incorporated nanoparticles designed to regulate calcium homeostasis have recently emerged as promising tools for modulating the bone microenvironment and attenuating pathological progression [119]. Similarly, selenium nanomodulation may be used to treat muscle diseases one day.

### 6.2. Synergistic Effects of Selenium with Other Trace Elements

Vitamin E (α-tocopherol) deficiency commonly coexists with selenium insufficiency. Notably, combined selenium and vitamin E deficiency serves as the predominant etiological factor in most documented myopathic syndromes. Experimental evidence indicates that concurrent selenium and vitamin E deficiency triggers systemic fatal myopathy in guinea pigs, whereas isolated deficiency of either micronutrient leads to attenuated pathological severity [120]. Lipid peroxidation was detected in affected muscle tissues concomitant with reduced GPx activity—a biomarker likely indicative of selenium deficiency. Furthermore, calves fed a selenium-deficient diet supplemented with adequate tocopherol and low polyunsaturated fatty acids [121] exhibited no myopathic manifestations. These findings suggest vitamin E may exacerbate the pathological consequences of selenium deficiency, though the mechanistic interplay remains unresolved [30]. Synergistic micronutrient supplementation strategies combining selenium and vitamin E have been employed to enhance antioxidant enzyme activity and improve endurance performance. Recent clinical trials demonstrated that the co-administration of selenium (17.5 μg/day as sodium selenite) and vitamin E (400 IU/day) significantly lowered post-exercise malondialdehyde (MDA) and lactate dehydrogenase (LDH) levels while elevating superoxide dismutase (SOD) and GPx activities compared to placebo (*p* < 0.05) [101,102,103].

### 6.3. Gut-Muscle Axis and Regulation of Selenium

Given that dietary selenium supplements are ingested and absorbed in the intestine, a gut microbiota–muscle axis may mediate selenium’s effects on skeletal muscle health. This axis potentially links microbial communities to exercise performance, muscle strength maintenance, and the prevention of motor function decline. Emerging evidence indicates that microbiota–muscle crosstalk and microbiota–mitochondrial communication modulate age-related muscle dysfunction. Specifically, the gut microbiota–muscle axis operates via the modulation of metabolic pathways and mitochondrial network dynamics in skeletal muscle, with dynamin-related protein 1 (DRP1)—a key regulator of mitochondrial fission—being essential to mitigate exercise capacity deterioration during aging [122].

## 7. Conclusions

Selenium plays a multifunctional role in preserving skeletal muscle homeostasis and enhancing exercise capacity through three primary mechanisms: antioxidative defense mechanisms, the precise regulation of selenoprotein biosynthesis, and the orchestrated modulation of redox-sensitive signaling pathways. Current mechanistic investigations remain predominantly confined to preclinical animal models, highlighting an imperative need for well-designed human clinical trials to validate these preclinical findings and establish causal relationships. The strategic integration of optimized selenium supplementation with evidence-based exercise protocols emerges as a promising intervention for mitigating age-related muscle decline and preventing exercise-induced musculoskeletal injuries. Future research priorities should focus on three translational dimensions: (1) dose–response optimization through pharmacokinetic/pharmacodynamic modeling, (2) systems-level elucidation of selenoprotein–muscle interactome networks, and (3) clinical validation of selenium’s ergogenic potential via randomized controlled trials, ultimately enabling targeted applications in sports nutrition and preventive medicine.

## Figures and Tables

**Table 1:** Functional summary of skeletal muscle-related selenoproteins.

Selenoprotein	Main Function	Regulatory Pathways/Molecular Mechanisms	Pathological Association	Subcellular Localization	References
SELENOW	Regulates calcium homeostasis, inhibits ubiquitin-proteasome degradation, and promotes muscle tube fusion Protects proliferating myoblasts from the influence of oxidative stress	RAC1-mTOR	Selenium deficiency myopathy and muscular dystrophy	Cytoplasmic	[19,27]
SELENOT	Maintains the redox balance of ER, inhibits apoptosis and autophagy	CHOP/ATF/SERCA2/NRF1/PGC-1α PACAP	Muscle aplasia, sports injuries	ER	[53,54,55,56,57]
SELENON	ER redox and calcium homeostasis	RyR1/SERCA	Congenital myopathy (multiple microaxonopathy)	ER	[21,26,27,58,59,60]
SELENOK	Participates in repair after skeletal muscle injury	Promote satellite cells-mediated myogenic differentiation; Prevents intracellular antioxidant dysfunction, apoptosis and autophagy by regulating the level of ER stress	Inhibition of skeletal muscle regeneration after injury	ER, plasma membrane	[10]
SELENOP	Selenium transporters maintain systemic selenium homeostasis and enhance antioxidant defense	ApoER2/LRP1 receptor-mediated selenium uptake	Selenium deficiency leads to muscle atrophy and age-related muscle loss	Secreted, cytoplasmic	[12,61,62]
SELENOS	Regulates endoplasmic reticulum-associated degradation (ERAD) to improve insulin sensitivity	SEL1L-HRD1 compound	Skeletal muscle injury and growth retardation due to ER stress	ER	[12,63,64,65]
GPx1	Removes cytoplasmic hydrogen peroxide (H_2_O_2_) to protect cell membrane integrity	Nrf2/HO-1; Inhibit oxidative stress and apoptosis; Correct insulin resistance; Regulate fatty acid metabolism;	Oxidative injury after exercise, chronic inflammation	Cytoplasmic	[27,66,67]
GPx4	Specifically inhibits lipid peroxidation and prevents iron death	Lipid peroxidation/ACSL4	Iron death muscle fiber necrosis	Cytoplasmic	[39,68]
SELENOH	Promotes DNA repair and maintains genomic stability	Unknown (possibly related to oxidative damage repair)	DNA damage and muscle fiber degeneration caused by selenium deficiency	Nuclear	[69]
DIO2	Catalyzes the conversion of T4 to T3, regulates thyroid hormone metabolism and energy balance	Thyroid hormone signaling pathway	Metabolic disorders after exercise, muscle fatigue	Membrane-associated	[70,71]
TXNRD1	Reduction-oxidized thioredoxin, repairs oxidation-damaged protein	Trx/ASK1	Chronic oxidative stress, muscle fibrosis	Cytoplasmic, nuclear	[41,42,43,44,45]
SELENOM	Maintenance of ER homeostasis; regulates glucose metabolism and insulin signal, affects muscle cell energy homeostasis	PI3K-Akt/mTOR	Skeletal muscle injury and growth retardation due to ER stress.	ER	[12,51,72]

**Table 2:** Summary of Human Trials on Selenium Supplementation and Exercise Performance.

Forms of Se	Dose	Intervention Duration	Sample Size	Outcome	References
selenomethionine	180 μg/d	10 weeks	24	No effect on the adaptation induced by endurance training	[97]
selenomethionine	240 μg/d	10 weeks	24	Increased the muscle GPx of subjects during acute exercise	[38]
sodium selenite	200 μg/d (Zinc 30 mg/d)	4 weeks	32	Simultaneous and individual supplementation of selenium and zinc had no significant effect on the resting testosterone and lactic acid levels	[98]
sodium selenite	200 μg/d	3 weeks	20	Reduced blood levels of lipid hydroperoxide postexercise in overweight adults	[77]
sodium selenite	17.5 μg/d (Vitamin E 400 IU)	3 weeks	8	Significantly improved endurance exercise performance (VO2max, AT, and endurance performance time), but decreased lactate dehydrogenase (LDH)	[100]
sodium selenite	17.5 μg/d (Vitamin E 400 IU)	3 weeks	9	SOD and GPx were significantly increased and MDA was decreased	[101]
sodium selenite	17.5 μg/d (Vitamin E 400 IU)	3 weeks	10	SOD and GPx were significantly increased, MDA was decreased. Cardiopulmonary endurance (VO2max, AT) was significantly increased	[102]
sodium selenite	17.5 μg/d (Vitamin E 400 IU)	3 weeks	10	SOD and GPX were significantly increased, but MDA was decreased. Vitamin E and selenium significantly reduced blood fatigue factors (NH3, LDH and phosphorus)	[103]
selenium tablets(Not mentioned)	200 μg/d	2 weeks	20	Reduced oxidative stress caused by physical exercise	[104]
